# Quantification of Protozoa and Viruses from Small Water Volumes

**DOI:** 10.3390/ijerph120707118

**Published:** 2015-06-24

**Authors:** J. Alfredo Bonilla, Tonya D. Bonilla, Amir M. Abdelzaher, Troy M. Scott, Jerzy Lukasik, Helena M. Solo-Gabriele, Carol J. Palmer

**Affiliations:** 1 Oceans and Human Health Center, University of Miami, Key Biscayne, FL 33149, USA; E-Mails: tdbonilla@mmm.com (T.D.B.); amabdelzaher@gmail.com (A.M.A.); drtmscott@gmail.com (T.M.S.); hmsolo@miami.edu (H.M.S.-G.); carolpalmer@biostarconsulting.com (C.J.P.); 2 Department of Biology, University of Wisconsin-River Falls, River Fall, WI 54022, USA; 3 3M Corporate Research Materials Laboratory, St. Paul, MN 55144, USA; 4 Department of Civil, Arch., and Environmental Engineering, University of Miami, Coral Gables, FL 33126, USA; 5 Hydros Coastal Solutions, Inc.-Miami, FL 33126, USA; 6 BCS Laboratories, Inc., Gainesville FL 32609, USA; E-Mail: Lukasik@microbioservices.com; 7 BioStar Consulting, Inc., Greenbrier, TN 37073, USA

**Keywords:** *Cryptosporidium*, *Giardia*, enterovirus, quantitative PCR, water quality

## Abstract

Large sample volumes are traditionally required for the analysis of waterborne pathogens. The need for large volumes greatly limits the number of samples that can be processed. The goals of this study were to compare extraction and detection procedures for quantifying protozoan parasites and viruses from small volumes of marine water. The intent was to evaluate a logistically simpler method of sample collection and processing that would facilitate direct pathogen measures as part of routine monitoring programs. Samples were collected simultaneously using a bilayer device with protozoa capture by size (top filter) and viruses capture by charge (bottom filter). Protozoan detection technologies utilized for recovery of *Cryptosporidium* spp. and *Giardia* spp. were qPCR and the more traditional immunomagnetic separation—IFA-microscopy, while virus (poliovirus) detection was based upon qPCR *versus* plaque assay. Filters were eluted using reagents consistent with the downstream detection technologies. Results showed higher mean recoveries using traditional detection methods over qPCR for *Cryptosporidium* (91% *vs.* 45%) and poliovirus (67% *vs.* 55%) whereas for *Giardia* the qPCR-based methods were characterized by higher mean recoveries (41% *vs.* 28%). Overall mean recoveries are considered high for all detection technologies. Results suggest that simultaneous filtration may be suitable for isolating different classes of pathogens from small marine water volumes. More research is needed to evaluate the suitability of this method for detecting pathogens at low ambient concentration levels.

## 1. Introduction

Preventing the transmission of waterborne diseases remains one of the most significant challenges facing public health professionals [1,2]. The measurement of the etiologic agents of waterborne disease is a critical component in assessing transmission routes and remains a major challenge. Potable water is typically monitored for fecal indicator organisms to assess whether it is free of sewage-derived microorganisms and pathogens. Monitoring coastal waters using indicator organisms alone, however, may not prove as efficient in detecting whether microbial pathogens are present in beach waters [3,4,5]. Pathogens may enter the water directly from humans and animals swimming and recreating on the beach and may not correlate with fecal indicator bacteria [6,7,8]. Human pathogens may also enter the coastal ecosystem through discharges from wastewater treatment plants which have effectively reduced the level of fecal indicator bacteria but have not eliminated all human pathogens. This is an important point because it has been shown that some pathogenic microorganisms are often more resistant to disinfection than indicator bacteria and can cause infections at very low doses [9,10,11]. Moreover, the persistence of fecal indicator bacteria in beach sand and soil has been well documented and may represent a reservoir of indicator bacteria in the absence of human pathogens [6,12,13,14,15]. To this end, there is a need for developing monitoring systems and assays that recover and detect multiple types of pathogens simultaneously.

The protozoan pathogens *Cryptosporidium* and *Giardia* are among the five most common etiologic agents of waterborne disease outbreaks in the US [16]. *Cryptosporidium* and *Giardia* have a low infectious dose and are very resistant to common disinfection procedures, particularly chlorination [10,17]. Between January 2007 and December 2008 there were a total 134 outbreaks associated with recreational water. In 60 of the 134 (44.8%), *Cryptosporidium* was identified as the etiological agent and resulted in 12,154 cases of cryptosporidiosis [18]. Moreover, the number of total cases of cryptosporidiosis and giardiasis reported to the U.S. Centers for Disease Control and Prevention (CDC) in 2011 was 9250 and 16,747, respectively [19]. Worldwide they are the most common protozoan parasites causing waterborne disease outbreaks [20].

Similarly, it has been demonstrated that many enteric viruses capable of causing disease in humans survive standard disinfection processes at wastewater treatment facilities [21]. Human enteric viruses are significant etiological agents of recreational waterborne illnesses. However, waterborne enteric viruses have historically been difficult to quantify due to their relatively low abundance and difficulties in culturing. Moreover, viral agents have been identified as the etiological agent in 7% of the recreational waterborne outbreaks in the US [16], and are suspected in 29% of the outbreaks caused by unknown etiological agents [22,23]. Adding to the challenges, human enteric viruses have been shown to be present in recreational water regardless of the concentration of fecal indicator bacteria (FIB) [17]. The lack of correlation between pathogens and fecal indicator bacteria has been attributed to effects of sunlight, differential growth and survival of indicators in the environment and hydrogeological variability [24,25]. The percent contribution of sewage effluent to the sample has also been found to be a variable impacting the ratios between pathogens and FIB [26].

The detection of pathogenic microorganisms from coastal water samples utilizing currently available methods is technically challenging. FIB may be detected after filtration of 100 mL of water through a membrane filter and then placement of the filter on media selective to culture the organism of interest [27]. Viruses are more difficult to detect and normally require the filtration of larger volumes of water (10-100 liters) through a specialized filter followed by chemical measures necessary to elute the viruses from the filter or membrane [27,28]. Protozoan pathogens typically are enumerated using USA EPA method 1623, which requires an alternative filter and elution method [27]. Molecular techniques, such as quantitative PCR (qPCR), have been developed to allow for the detection and identification of multiple microorganisms to the species (and even strain) level; however, before these techniques can become commonplace in pathogen monitoring they must be easy to measure and vigorously tested to assure their usefulness in addressing water quality and public health issues.

Although analytical methods for detection of pathogens are available, sample collection and concentration represents a major barrier in transitioning from FIB measures towards direct measures of pathogens. The costs and logistics are more difficult if larger samples are needed. Available sample concentration methods include hollow-fiber ultrafiltration [29,30]. This is a promising technique which has been evaluated for its ability to effectively recover bacteria, viruses and protozoa collectively [31,32,33,34]. Similarly electro-negative membrane-vortex methods [35] have been shown to be promising for sample concentration. Sassoubre *et al.* 2012 [36] observed promising recoveries of enterovirus using a flocculation-based skim milk method and dead-end membrane filtration. A simple bilayer filtration device has also been developed for the simultaneous capture of enterococci and coliphage in one filtration process [37]. This bilayer device consisted of two filters (0.45 µm pore size, 90 mm diameter) in series separated by a 1 cm spacer. The top filter consisted of a low protein binding membrane (PVDF, Millipore, Durapore-hydrophilic) which retaind microbes based upon size exclusion (*i.e.*, the protozoa). The lower filter was a high protein binding membrane with a slight negative charge (HA, Millipore, MF-Millipore) which permited for the binding of viral particles which tend to be positively charged in marine waters at near neutral pH and low pH values [37,38]. Water was drawn through the filter by applying a vacuum. The advantage of the bilayer device is that it avoids the need to split the concentrates as two filters are produced from a simultaneous filtration process, one for protozoa and another for virus analyses. Ultrafiltration and vortex methods require the split of the concentrates if both protozoa and viruses are to be evaluated, thus requiring twice the sample volume. The bilayer device thus increases the effective sample volume by two per unit water filtered which simplifies the logistics of sample collection. This bilayer filtration device, however, has not been tested with pathogens. The objective of the current study was to evaluate the simple bilayer filtration device in its ability to recover protozoan (*Cryptosporidium* and *Giardia*) and viral (poliovirus) pathogens, followed by detection using either qPCR or traditional detection technologies. Additionally, new filter elution and DNA extraction methods compatible with the detection technologies were developed in order to process the concentrates from the device for these pathogen measures. This work is significant because it contributes towards the scientific knowledge base in pursuit of developing methods that will facilitate effective and precise routine measurement of pathogens in the environment.

## 2. Experimental Section

### 2.1. Sample Collection and Preparation

Three trials were run. Either 15 liters (Trial A and B) or 30 liters (Trial C) of ocean water (~ 4.5 NTU/salinity 36 ppt/pH = 8/ temperature 30 ºC) were collected on three different occasions from knee-deep water at a beach located in Miami-Dade County, Florida, USA. Five (Trial A and B) or 10 (Trial C) liter portions of the ocean water were spiked with 200 µL suspensions of live *Giardia lamblia* cysts and live *Cryptosporidium parvum* oocysts. One milliliter suspensions of live poliovirus were spiked into each 5 liter or 10 liter seawater sample. For *Crytosporidium* and *Giardia* analyses, Trial A was run in duplicate, Trial B in triplicate, and Trial C in quadruplicate for the qPCR portion only. Poliovirus by qPCR was run in duplicate for Trial B and quadruplicate in Trial C. Poliovirus by plaque assay was run in triplicate in Trial C. The plaque assay for poliovirus for Trial B was omitted due to problems with the cell line supporting the growth of poliovirus. The IMS/IFA was omitted from Trial C due to budgetary constraints.

Suspensions of live poliovirus (CHAT strain, ATCC VR-1562) were propagated in-house using Buffalo Green Monkey (BGM) cells and stock suspensions used for spiking seawater were measured at 10^6^ PFU mL^−1^ and stored in the refrigerator until needed. Immediately prior to experimentation an aliquot of the stock solution was diluted to 10^3^ PFU mL^−1^ and these dilutions were used to spike the seawater samples. The *G. lamblia* cysts and *C. parvum* oocysts were purchased from the Wisconsin Department of Health laboratory and were certified to contain 500 cysts per 200 µL of viable *Giardia* and 500 oocysts per 200 µL of viable *Cryptosporidium*. Due to the relatively low numbers of protozoa in the stock suspensions they were not further diluted and were used directly to spike the seawater. Spiking suspensions from each trial were analyzed using the corresponding analytical technique in order to obtain a direct measure of the initial microbe concentration. For example, for Trial A the protozoa spiking suspension was split and analyzed by qPCR and by IMS/IFA. The values of the initial concentrations for the corresponding method were then compared to the microbes recovered from the filters to compute percent recoveries. The basis of comparison was set to 1 mL equivalent of the spiking solution for poliovirus and 200 µL of the spiking solution for protozoa (Table 1). Although the target concentrations of the spiking solutions was 10^3^ PFU per mL for poliovirus and 500 oocysts or cysts per 200 µL for the protozoa, the actual initial microbe concentrations differed and were lower than the target values. Actual concentrations of the target microbes were generally lower when measured by culture or microscopic methods as compared to qPCR.

For the seawater used for filtration, the target concentration of the protozoans was 50 to 100 cysts/oocysts per L of seawater. The target concentration of poliovirus in the seawater used for filtration ranged from 100 to 200 PFU per L. The actual concentrations in the seawater given the reductions observed in the spiking solutions was less than the target value and varied by analysis method and trial number. This may have been caused by die-off within the spike solutions or the effects of indigenous microbes within the seawater samples. Blank filters (no spike) were run for each trial and the eluates for all trials showed levels of poliovirus and protozoa that were below the limits of detection.

**Table 1:** Initial Concentration of Spiking Solutions as Measured for each Trial. Target concentrations of the spiking solutions were 500 oocysts or cysts per 200 µL for *Cryptosporidium* or *Giardia* and 10^3^ per mL for poliovirus.

Trial	*Cryptosporidium* per 200 µL		*Giardia* per 200 µL		Poliovirus per mL	
	qPCR	IMS/IFA	qPCR	IMS/IFA	qPCR	Plaque Assay
A	240	232	184	128	−	−
B	372	102	367	262	634	−
C	452	−	384	−	834	240

### 2.2. Filtration Setup and Procedure

The water samples were mixed vigorously and filtered through the bilayer filtration device designed to collect protozoa on the top filter, and viruses on the bottom filter. The custom-made stainless steel 90 mm diameter filtration device allows two 90 mm membranes to be placed one over the other, with enough space in between (approximately 1 cm) to prevent the pores of the membranes from overlapping [37]. Two types of 0.45 µm pore size, 90 mm membranes were used in this study. For the top, a polyvinylidene fluoride (PVDF) membrane (Millipore, Durapore-hydrophilic) characterized by low protein binding, was used. The PVDF membrane has been shown to permit the passage of viruses but retain bacteria [38]. A high protein binding type HA membrane with a mixed cellulose ester composition (Millipore, MF-Millipore) was used as the bottom membrane. The properties of this membrane allow for the adsorption of viruses which permeate through the top membrane [27,38,39,40].

### 2.3. Elution and Analysis of Protozoa from Top Membrane

Consistent with the FiltaMax™ (IDEXX) method the top membrane was not allowed to completely dry with about 20 mL concentrate allowed to remain. This concentrate containing the entrapped protozoa was drawn off and placed into a sterile 50 mL conical tube. The top membrane was then eluted with 10 mL followed by 5 mL of phosphate buffered saline (PBS) +0.01% Tween 20 solution adapted from the FiltaMax™ (IDEXX) method. This was done by placing the membrane in a sterile Whirlpack™ bag and rubbing for 3 minutes then adding the eluent to the volume of concentrate drawn off the top filter. The total eluent (35−50 mL) was split for processing for qPCR analysis and IMS/IFA analysis.

For qPCR analysis, the eluant was concentrated using a 10-kDa MWCO Amicon-15 (Millipore) ultra-filtration tube at 3000 × g until volume was below 300 μL. This required multiple additions of eluent in some cases. The sample concentrate was processed for DNA extraction with the MoBio Power Soil DNA Isolation Kit according to the manufacturer’s instructions with slight modification. Briefly, after the addition of the lysis reagent, proteinase K (100 µg mL^−1^) was added and the sample was incubated for 1 h at 65 °C followed by 7 cycles of freeze-thaw in liquid nitrogen/65 °C water bath for 5 min each. Following the freeze-thaw cycles, the instructions from the kit were resumed and the DNA was eluted in 60 μL dH_2_O.

For direct concentration and enumeration of parasites using IMS/IFA microscopy, U.S. EPA Method 1623 was followed. Briefly, this method involves purification using immunomagnetic separation (Dynal IMS beads GC Combo) followed by application of a fluorescent antibody stain (EasyStain, Biotech Frontier) and analysis under an epifluorescence microscope [41].

### 2.4. Elution and Analysis of Viruses from Bottom Membrane

The processing of the bottom filter for viruses also required the development of an appropriate elution technique. To this end, one set of bottom filters was prepared for Trial B and two sets of bottom filters were prepared for Trial C. The bottom filters prepared from Trial B and one set of bottom filters from Trial C were processed for qPCR analysis and the second set of bottom filters from Trial C were process for plaque assay. For qPCR, the viruses trapped on the bottom filter were eluted with 10 mL of guanidinium hydrocloride detergent solution (AL/ASL solution in a 1:1 ratio from the Qiagen Blood kit plus 0.1 mL of Antifoam A per 120 mL of elution solution to prevent foaming). Total RNA was isolated using the Qiagen Blood kit according to the manufacturer’s instructions. The RNA was further processed using Trizol reagent (Invitrogen) and re-suspended in a final volume of 40 µL.

For cell culture plaque assay, the bottom filter was eluted with 10 mL of 0.1% Tween 80 in PBS. Poliovirus was enumerated as plaque forming units. Briefly, viruses in 100 µL of the sample concentrate from the bottom filter was inoculated on freshly prepared monolayers of BGM cells and plaque assays were performed using 2X dMEM (MediaTech, USA) and 2X Bacto Agar containing 0.0001% Neutral Red. Cell flasks were incubated at 36.5 °C for 72 h. Plaques on the respective flasks were counted and percent recoveries were calculated.

### 2.5. Reverse Transcription and Quantitative PCR Analysis

RNA (6 µL) was reverse transcribed with random hexamers using the cDNA First Strand Synthesis kit (Invitrogen). The cDNA was subsequently analyzed for poliovirus via qPCR.

Quantitative PCR was performed using TaqMan probes that add to the specificity of the amplification reaction, as it requires additional complementary sequences within the 2 primer-binding locations. The primers and probes used to detect *Giardia*, *Cryptosporidium* [42] and poliovirus [43] are listed in Table 2. Initially, gene targets were amplified by PCR from stock controls and the amplicons were cloned into pCR4 plasmid (Invitrogen) and sequenced for confirmation. The plasmids were purified with Qiagen Mini-prep columns and the DNA concentration was determined by UV spectrophotometry. The concentration of DNA was used to determine the copy number of gene targets to generate a standard curve for absolute quantification of target gene copies. The standard curves were generated from serial 10-fold dilutions of the control plasmids and were prepared from a preserved stock for each experiment. To control for inhibitory substance that may have remained in the DNA preparations of the top filter, exogenous DNA was added to each sample prior to qPCR analysis. For the RNA extracts of the bottom filter, exogenous RNA was added immediately prior to the cDNA synthesis reaction. Primers and probe specific to the control nucleic acid were used in a qPCR reaction and the Ct value obtained from each environmental DNA or RNA sample was compared to a water-control sample. Typically, the described protocol demonstrated the absence of inhibitory agents in the nucleic acid preparations, or a measure of the level of inhibition was determined.

**Table 2:** Primers and Probes used in this study.

Target *(Ref.)*	Sequence 5′–3′
*Giardia* spp. [42]	
Primer G101	CATCCGCGAGGAGGTCAA
Primer G102	GCAGCCATGGTGTCGATCT
Probe G103	6FAM-AAGTCCGCCGACAACATGTACCTAACGA-IB
	
*Cryptosporidium* spp. [42]	
Primer C104	CAAATTGATACCGTTTGTCCTTCTG
Primer C105	GGCATGTCGATTCTAATTCAGCT
Probe C106	6FAM-TGCCATACATTGTTGTCCTGACAAATTGAAT-IB
	
Poliovirus [43]	
Primer P107	CCTCCGGCCCCTGAATG
Primer P108	ACCGGATGGCCAATCCAA
Probe P109	6FAM-CGACTACTTTGGGTGTCCGTGTTTCC-IB
	
Internal control nucleic acid (This study)	
Primer P110	CATGATAAGGTTTTGAGCTCTGTGTATTG
Primer P111	TCCTTTTTGTGCATAACCTGATTTAA
Probe P112	6FAM- ACATATGTAAAAGAGAGCTTC-MGBNFQ

A standard curve covering an 8-log titration of the control plasmids carrying each gene target (COWP gene of *Cryptosporidium* spp., β-giardin gene in *Giardia* spp. and 5′ non-translated region of poliovirus) was successfully generated for absolute quantification of target gene copies in the concentrated water sample. The standard curve was prepared from aliquot stocks of control plasmids for each run and the slope and regression of the curve was analyzed. The slope of each qPCR standard curve is an indication of the efficiency of the qPCR reaction. Plotting Ct *versus* log_10_ of gene copies in the template DNA yields a line with a slope of −3.32 for a reaction with 100% efficiency (Slope = −1/log_10_ 2 = −1/0.301 = −3.32). The qPCR reaction was considered successful and the data analyzed only if the slope was between −3.10 and −3.58. For all standard curves the coefficient of correlation (R^2^) > 0.99.

While the efficiency of the standard curve can be calculated based on the titration of a known amount of plasmid copies, for the experimental water samples inhibition within the amplification tube was evaluated. To do so, we seeded every qPCR reaction with an equal amount of exogenous nucleic acid. We used DNA and RNA from *Plasmodium falciparum*, an intracellular human pathogen not expected to be in environmental samples. The control DNA was short target fragments added into each qPCR reaction for *Giardia* and *Cryptosporidium* and amplified concomitantly. The Ct value was checked against a water control to determine whether inhibition was present in each environmental DNA sample. For the analysis of environmental RNA samples, *in vitro* RNA transcripts synthesized using a T7 RNA polymerase transcription reaction were seeded into each cDNA synthesis reaction. Again, the Ct value was checked against a water control to determine whether inhibition was present in each environmental RNA sample. The quantification of seeded nucleic acid in the control and the experimental samples were nearly identical (+/−3%) (data not shown). It was determined that this variability was due to the technical limitations of qPCR and qRT-PCR and that the environmental samples demonstrated no inhibition.

## 3. Results

*Cryptosporidium* and *Giardia* seeded into ocean water and passed through the bilayer filtration device were captured, eluted, and detected by both qPCR and IMS/IFA assays. Three independent experiments were performed for a total of 9 data points for qPCR and 5 replicates were performed for IMS/IFA. Similarly poliovirus bottom filters were analyzed for a total of 6 data points for qPCR and 3 for plaque assay. Results are representative of the combined methods that incorporate the small volume bilayer concentration method with the different elution/extraction methods coupled with the different detection technologies. Variability can be noted in the results. This variability could not be attributed to known errors and is incorporated into the overall statistical analyses.

For *Giardia* cysts, mean recovery by qPCR was 41% with a standard deviation (σ) of 26% in comparison to 28% (σ of 11%) for IMS/IFA. For *Cryptosporidium* oocysts, mean recovery by qPCR was 45% (21%) in comparison to 91% (39%) for IMS/IFA (Table 3). Statistical analysis using a paired t-test (Sigma Stat) demonstrated that the differences in *Giardia* cyst recoveries were not statistically significant (*p* > 0.10). *Cryptosporidium* oocysts recovery was higher for IMS/FA relative to qPCR (*p* = 0.03).

**Table 3:** Percent Recovery of *Cryptosporidium* oocysts and *Giardia* cysts by qPCR *vs.* IMS/FA. Recoveries incorporate differences in extraction and detection methodologies.

Trial/Replicate	Percent Recovery			
	*Cryptosporidium*		*Giardia*	
	qPCR	IMS/IFA	qPCR	IMS/IFA
A1	55	61	98	34
A2	51	52	49	38
B1	23	116	40	34
B2	39	145	2	20
B3	22	83	61	13
C1	85	−	16	−
C2	56	−	48	−
C3	52	−	25	−
C4	23	−	31	−
Average	45	91	41	28
Std. Deviation	21	39	26	11

For poliovirus, the qPCR method resulted in a mean recovery of 55% (37%) after the bilayer filtration (Table 4). The plaque assay resulted in a mean recovery of 67% (22%) of poliovirus plaque-forming units. Statistical analysis of these data using a paired t-test analysis demonstrated that the difference in recovery between the qPCR and plaque assay methods was also not statistically significant (*p* > 0.10). While the mean recoveries for all organisms by both methods are encouraging, high variability was observed.

**Table 4:** Percent Recovery of Poliovirus qPCR *vs.* plaque assay. Recoveries incorporate differences in extraction and detection methodologies.

Trial/Replicate	Poliovirus Percent Recovery	
	qPCR	Plaque Assay
B1	86	−
B2	106	−
C1	13	75
C2	68	41
C3	22	83
C4	34	
Average	55	67
Std. Deviation	37	22

## 4. Discussion

There is a need to increase our understanding of the complex interactions between ocean health and human health. Worldwide, nearly 60% of the world’s population is estimated to live in coastal areas [44] and assessing the impacts of these populations on coastal water quality is a necessary first step in developing sustainable coastal ecosystems. While there are many environmental risk factors associated with negatively impacted coastal ocean water, human pathogens remain the major public health concerns. The current methods of assessing the hygienic quality of coastal ocean water remain inadequate in addressing this important concern. This is because indicator organisms have been shown to persist and multiply in the environment making their predictability of recent contamination questionable [13,45]. However, the direct detection of pathogens is highly time consuming by conventional microbiological techniques.

Advances in molecular microbiology have led to the creation of methods capable of detecting multiple organisms simultaneously. While limitations exist in genetically identifying microorganisms in the environment, it is generally accepted that methods will continue to be improved in order to address these limitations. The development of assays capable of identifying multiple organisms, including different classes of pathogens, will be extremely valuable for water quality and public health professionals and could outweigh some of the limitations these methods present. To improve upon these methods, the development of new assays needs to be thoroughly tested against traditional standard detection methods in order to get a better understanding of their efficiency.

In this study, we demonstrated the ability to simultaneously recover protozoan and viral organisms from ocean water. The concentrates for protozoa and viruses corresponded to the exact same sample as opposed to a sample split. Bilayer filtration allowed for reduction of the volume of the original water sample by a factor of two, which in turn simplified sample collection. In addition, we compared molecular methods of identifying two medically important protozoan pathogens and a representative enterovirus (the CHAT strain of poliovirus) to more conventional, and often time-consuming, methods. The methodology successfully captured the different types of organisms onto their respective membrane types demonstrating that our method is feasible with actual live pathogenic protozoans and viruses. The pathogen extraction and detection efficiencies from the filters were relatively high and were comparable to those achieved using conventional methods. For example, Francy *et al.* [46] found between 40 and 60 percent recovery for *Cryptosporidium* oocysts and *Giardia* cysts (given spikes of 10 oocysts or cysts per liter, equivalent to 1000 per 100 L) and much lower recoveries for enteroviruses (<15%) for spikes of 8.4 x 10^5^ per liter. Similarly, Keserue *et al.* [47] measured 13% and 30% recoveries for *Cryptosporidium* and *Giardia*, respectively, for spikes consisting of a couple thousand oocysts or cysts per liter. Haramoto *et al.* [35] recovered between 28 and 87% of poliovirus, and 23% and 60% of *Cryptosporidium* and *Giardia*, respectively.

Although mean recoveries of poliovirus and *Cryptosporidium* were higher using traditional detection technologies in this study, a major advantage of the molecular assay is that it was faster. The amount of time to run PCR is on the order of 4 hours. However, analysis of protozoa by microscopic examination can take upwards of 1 day whereas plaque assays for viruses can take up to many days. Another advantage of PCR is its ability to be easily adapted to detect additional pathogens leading to a rapid and more thorough analysis of the microbial content of water. The efficiency of recovery for the organisms tested was comparable with traditional large-volume methods currently used to identify the organisms in recreational water. Moreover, the combined methods of filtration, elution, extraction, and detection were also capable of recovering high proportions of pathogenic microbes. The recovery and quantification using the bilayer filtration device and qPCR was significantly more rapid (hours) than the traditional methods of immunomagnetic separation/immunofluorescence for the protozoan pathogens *Giardia* and *Cryptosporidium*, and plaque assay for enteroviruses (day to many days). As molecular detection protocols are developed and validated for additional microorganisms, this technical advancement could add to the development of methods to change the way we monitor water quality to ensure human health and safety. This study demonstrated, at the pilot scale, the promise of recovering and detecting pathogens from small volumes of water using the described filtration, elution, extraction, and detection technologies.

We recommend further study to improve recovery and to minimize variability. More testing is needed to evaluate recoveries at lower concentrations, at levels typically observed within recreational waters. In the current study the levels of poliovirus used during experimentation were 10,000 to 20,000 per 100 L. This is high in comparison to non-sewage-impacted recreational waters for which enterovirus levels are in the 2 to 9 MPN per 100 L range [28,48]. The levels used during experimentation are more consistent with levels observed in sewage-impacted waters. For examples levels by qPCR have been observed as high as 12,500 genomic equivalents per 100 L for a sewage-impacted watershed in South Africa [49], and 450,000 genomic equivalents per 100 L for sewage-impacted beaches in Ohio, USA [50]. Similarly for *Cryptosporidium* and *Giardia*, the experimental levels used in the current study are high (5000 to 10,000 oocysts/cysts per 100 L) in comparison to levels observed in environmental samples. In North American surface water *Cryptosporidium* levels have been observed at 43 to 270 oocysts per 100 L on average [10,51,52] and *Giardia* levels at 3 to 280 cysts per 100 L on average. Sewage impacted surface waters in Chicago were measured at 1 *Cryptosporidium* oocyst per 100 L and 65 *Giardia* cysts per 100 L. At the very high end, impacted Venezuelan beaches have measured *Cryptosporidum* at 200 oocysts per 100 L and *Giardia* 1700 cysts per 100 L [53]. The numbers in Malaysia are similar to that in Venezuela measuring at up to 233 oocysts per 100 L and 400 cysts per 100 L [54]. These high levels observed in sewage-impacted waters are still low in comparison to the levels evaluated in this study. More research is needed to evaluate recoveries at the lower levels typically observed in environmental samples.

## 5. Conclusions

The bilayer system utilized in this study was capable of simultaneously capturing protozoan pathogens by size exclusion and poliovirus by charge. More research is needed to evaluate the system at lower concentration levels and to minimize the variability in percent recoveries. Upon further study we envision the utilization of this and similar technologies for measurements of pathogens as part of routine monitoring programs. Direct measures of pathogens would improve the ability to assess potential health effects of waterborne contaminants, thereby helping to remove the technological gap in processing water samples for pathogen analyses.
